# Socio-Behavioral Characteristics of Parents/Guardians Associated with Child Dental Neglect: A Retrospective Cross-Sectional Analytical Study

**DOI:** 10.3390/children13060801

**Published:** 2026-06-10

**Authors:** Anamaria Violeta Țuțuianu, Dan Alexandru Slăvescu, Abel Emanuel Moca, Teodora Ștefănescu, Lucian Roman Șipoș, Horia Câlniceanu, Anca Ionel

**Affiliations:** 1 Department of Dentistry, Faculty of Medicine and Pharmacy, University of Oradea, 4 Universității Street, 410087 Oradea, Romania; anamaria.tutuianu@uoradea.ro (A.V.Ț.); slavescudan@uoradea.ro (D.A.S.); tstefanescu@uoradea.ro (T.Ș.); lsipos@uoradea.ro (L.R.Ș.); 2 Anton Sculean Research Center for Periodontal and Peri-Implant Diseases, Department of Periodontology, Faculty of Dental Medicine Victor Babeș, University of Medicine and Pharmacy, 300041 Timișoara, Romania; calniceanu.horia@umft.ro; 3 Department of Oral Rehabilitation, Faculty of Dental Medicine, Iuliu Hațieganu University of Medicine and Pharmacy Cluj-Napoca, 400012 Cluj-Napoca, Romania; ionel.anca@umfcluj.ro

**Keywords:** child dental neglect, pediatric dentistry, socioeconomic status, child maltreatment, oral health

## Abstract

**Background and Objectives:** Child dental neglect is a clinically significant form of maltreatment that frequently reflects broader challenges related to caregiving within the family environment. Although oral manifestations have been described in prior research, the socio-behavioral profile of responsible caregivers remains insufficiently characterized, particularly in Central and Eastern European contexts. This study aimed to identify caregiver-level socio-behavioral characteristics associated with child dental neglect and to examine their relationships with clinical outcomes. **Materials and Methods:** A retrospective cross-sectional analytical study was conducted on 333 children (aged 4–17 years) diagnosed with dental neglect, presenting at a municipal hospital and a private dental practice in Oradea, Romania (2020–2024). Caregiver-level variables included age, educational attainment, socioeconomic status, health condition, substance use, and family structure. Associations were analyzed using Fisher’s Exact Test, Pearson Chi-Square, and Mann–Whitney U test, with Bonferroni correction applied where appropriate. **Results:** Most caregivers were young adults (93.1%), with low educational attainment (40.2% had no formal schooling) and high rates of alcohol use (47.1%). Low family income was present in 89.2% of cases and was significantly associated with non-adherence to the dental treatment plan (*p*  =  0.039). Caregivers without formal education were associated with neglect in rural areas (43.4% vs. 26.2%; *p*  <  0.001). Children of drug-using caregivers were significantly older at presentation (median: 12 vs. 8 years; *p*  =  0.014), and caregiver drug use was more prevalent in urban settings (18.0% vs. 1.8%; *p*  <  0.001). Over half of the children (52.9%) came from disrupted family environments. **Conclusions:** Dental neglect was consistently associated with young, poorly educated, and financially disadvantaged caregivers exhibiting high rates of substance use and unstable family structures. These factors may interact in complex ways, highlighting the multifactorial nature of dental neglect. Dental professionals are well positioned for early identification and have a professional and ethical responsibility to integrate child safeguarding into routine clinical practice.

## 1. Introduction

Dental neglect is a serious but often overlooked form of child maltreatment with significant consequences for pediatric health and development. It is officially defined as a parent’s or guardian’s intentional failure to seek or follow through with necessary treatment to maintain an oral health level that is essential for proper function and free from pain and infection. Unaddressed dental neglect can cause serious oral health issues, such as untreated dental caries, periodontal disease, and tooth-related infections, all of which can hinder long-term development, impair feeding, and cause chronic discomfort. Importantly, dental neglect should not be seen solely as a sign of socioeconomic hardship; it often points to broader issues of neglect or abuse within the home environment [1,2,3].

A thorough understanding of the behavioral and psychological profiles of caregivers of children with dental neglect is crucial for the timely identification and effective intervention in cases of child dental neglect. Caregivers of children with dental neglect may exhibit a range of traits, including controlling tendencies, emotional instability, a personal history of childhood abuse or neglect, and active substance misuse. Typical behavioral signs include minimizing or outright denying the child’s symptoms, repeatedly failing to attend medical or dental appointments, and showing a general indifference toward the child’s physical well-being. Recognizing these patterns, along with clinical signs of oral neglect, equips healthcare professionals with the knowledge needed to take appropriate protective actions for vulnerable children [3,4,5,6].

One of the most significant risk factors for child neglect is parental or caregiver substance misuse. A large body of evidence has confirmed a strong link between alcohol and illicit drug use and the failure to fulfill children’s basic physical, emotional, and medical needs, including those related to oral hygiene, nutrition, and healthcare access. Substance use disorders impair parental ability by affecting judgment, reducing impulse control, and causing emotional unavailability, together undermining the consistency and quality of caregiving. Children in households affected by substance dependence are more likely to face unsafe living conditions, insufficient supervision, and systematic denial of essential medical and dental care. The ongoing instability typical of such environments often leads to extended neglect, with documented long-term effects on developmental, psychological, and physical health outcomes [7,8,9,10,11].

An increasing body of evidence highlights the crucial role of socioeconomic factors, including poverty, low educational levels, unemployment, and limited access to healthcare, in influencing a caregiver’s ability to provide adequate oral healthcare for children. Families from lower socioeconomic backgrounds often face financial pressures that force them to prioritize basic needs like food and shelter over routine dental visits, preventive care, or prompt treatment of dental issues [12].

Oral health maintenance is often compromised in disorganized or dysfunctional family systems, where the boundaries of parental roles and responsibilities for providing basic needs are unclear or inconsistently enforced. In such cases, dental neglect may not be intentional but is more likely a result of broader family dysfunction that hinders the systematic delivery of adequate care across multiple areas [13].

Poverty is a significant social factor affecting child health in various ways, with dental neglect being one of its most serious clinical issues. Children from low-income families are particularly vulnerable because they often have limited access to dental care, low oral health literacy, and financial difficulties. Basic barriers to care, such as a lack of dental insurance, transportation issues, and a shortage of dentists willing to treat patients with or without public insurance, are common challenges faced by families in financial hardship. Moreover, the lack of oral health education prevalent in low-income communities often results in poor oral hygiene habits, which, combined with systemic access barriers, heighten the risk of dental neglect. Consequently, children in impoverished households are more likely to develop untreated dental problems, negatively impacting their long-term health, education, and overall quality of life [13].

Dental professionals often have a privileged role as early observers of neglect because clinical signs may first appear during routine oral exams. Important signs requiring increased clinical attention include extensive untreated cavities, poor oral hygiene, and a pattern of missed dental appointments. This role is further emphasized by epidemiological data showing that about 65% to 75% of child maltreatment cases involve injuries to the head, neck, or mouth, placing dental practitioners at the forefront of abuse detection within the healthcare system [2,3,4].

Effective management of child neglect requires a coordinated, interprofessional response. Dental practitioners are encouraged to collaborate with pediatricians, social workers, and, when appropriate, law enforcement agencies to ensure that all aspects of the child’s health, safety, and welfare are thoroughly evaluated and addressed. This multidisciplinary approach is vital for comprehensive child protection. Additionally, in an increasing number of jurisdictions, dental professionals are legally required to report suspected abuse or neglect to appropriate child protection authorities. This legal duty reflects a broader ethical responsibility to protect vulnerable children, and failing to comply can lead to serious legal consequences and continued harm to the child [14].

This study aimed to describe the socio-behavioral profile of parents and guardians linked to child dental neglect and to identify the socioeconomic, familial, and environmental factors potentially associated with its occurrence. Specifically, the study sought to outline the behavioral patterns of caregivers of children presenting with dental neglect and to analyze the social, economic, and family conditions that may lead to neglecting children’s oral health needs.

## 2. Materials and Methods

### 2.1. Study Design and Ethical Approval

A retrospective cross-sectional analytical study was conducted on a group of pediatric patients diagnosed with dental neglect. Ethical approval was obtained from the Research Ethics Committee of the Faculty of Medicine and Pharmacy at the University of Oradea (Approval No. CEFMF/08, 5 October 2020), and all procedures adhered to the ethical standards of the Declaration of Helsinki and its later revisions. Before enrollment, written informed consent was obtained from the parents or legal guardians of all participating children. In cases where dental neglect was suspected, the consent process was handled carefully to ensure voluntariness, confidentiality, and the separation of research participation from routine clinical care. The detailed methodological framework for the clinical assessment of the children has been described elsewhere in a previous publication [15].

### 2.2. Study Population and Eligibility Criteria

The target population included children who presented for consultation at the “Dr. Gavril Curteanu” Municipal Clinical Hospital—Pediatric Department and at a private dental practice in Oradea, Romania, over a four-year period from 2020 to 2024. Inclusion criteria were: age between 4 and 17 years; residence in Bihor County; parental or guardian consent for clinical examination and photographic documentation; and the presence of oro-dental lesions consistent with a diagnosis of dental neglect, as defined by the American Academy of Pediatric Dentistry [16]. Children were excluded if the diagnosis of dental neglect was uncertain, if oral findings were more consistent with isolated severe dental disease without supporting contextual evidence of neglect, if residence was outside Bihor County, or if parental/legal guardian consent was not obtained. Children were also excluded when incomplete medical records prevented reliable assessment of the diagnostic criteria. Children with co-existing mental health disorders or general medical conditions were not excluded, provided their oro-dental findings were considered clinically consistent with dental neglect, in accordance with the study aim of exploring relationships between systemic variables and neglect severity.

To reduce diagnostic subjectivity, classification was not based solely on the presence of severe untreated dental disease. Instead, a diagnosis of dental neglect required the concurrent presence of both clinical indicators and caregiver-related contextual indicators.

Clinical indicators included untreated cavitated carious lesions, poor oral hygiene, recurrent dental pain or infection, preventable complications (e.g., pulpal involvement, dentoalveolar abscesses, or fistulae), delayed presentation despite visible disease progression, and repeated missed follow-up appointments. Contextual indicators included caregiver awareness of the child’s oral condition, previous professional recommendations not followed, delayed or interrupted treatment-seeking behavior, and repeated failure to attend appointments despite explanation of the risks, treatment plan, and available treatment options.

To distinguish dental neglect from limited access to care alone, children were not classified as neglected solely on the basis of socioeconomic disadvantage, rural residence, or barriers to dental access. Cases were considered consistent with dental neglect only when unmet oral healthcare needs persisted despite caregiver awareness of the child’s condition and professional recommendation for treatment.

The final analytic sample consisted of 333 children. Since this was a retrospective cross-sectional analytical study based on the complete enumeration of all eligible cases identified during the designated study period, an a priori sample size calculation was not applicable; instead, all consecutive children meeting the inclusion criteria were included, thereby maximizing the available statistical power.

The baseline assessment included a thorough medical and dental history (obtained with caregiver help), a general physical exam, and a complete oro-dental evaluation covering both extraoral and intraoral inspection. It also involved digital radiography (Pax-i 3D Green, Vatech, Hwaseong, Republic of Korea) and standardized intraoral photographs (MD-1500A, Mouthwatch, Metuchen, NJ, USA). All clinical evaluations were performed by two calibrated dental practitioners (A.V.Ț. and A.E.M.) using a standardized diagnostic protocol, with findings systematically documented on structured data collection forms. To improve diagnostic consistency, the examiners aligned diagnostic criteria through joint review of representative clinical cases, radiographs, and intraoral photographs prior to study implementation. Radiographic and photographic materials were reviewed jointly, and ambiguous cases or disagreements were resolved through consensus discussions to minimize inter-observer variability. Due to the retrospective nature of the study, formal inter-examiner reliability statistics were not prospectively recorded.

In addition to the child-level clinical data detailed in the prior publication [15], this study also collected a complementary set of variables related to the parents or legal guardians responsible for the child’s care. The following caregiver-level data were recorded:

- Identification of the caregiver of children presenting with dental neglect (biological parent, legal guardian, or other caregiver);
- Mechanism by which neglect occurred;
- Caregiver’s age;
- Level of formal education attained;
- Socioeconomic status, determined by reported household income;
- General health condition;
- Use of alcohol and/or psychoactive substances;
- Family structure and type (intact/traditional versus disorganized family unit).

### 2.3. Variable Definitions

Clinical and systemic variables were defined as follows. Oral hygiene was considered good if there was no visible plaque or calculus deposits and the gingival tissue was clinically healthy; it was classified as poor when plaque or calculus were present along with gingival inflammation and/or bleeding [17]. Obesity was defined as a body mass index at or above the 95th percentile for age and sex, based on WHO growth reference charts [18]. Malnutrition was identified by an underweight appearance, signs of growth delay or muscle wasting, and/or caregiver-reported dietary insufficiency, in line with WHO guidelines [19]. Mental health status was assessed through caregiver report and direct clinical observation, and categorized as: (a) healthy, (b) intellectually disabled, or (c) substance user; no formal psychometric assessment was performed.

The severity of oro-dental lesions was categorized into three levels: minor (uncomplicated carious lesions limited to one or two teeth, without pulpal involvement or soft tissue inflammation); moderate (deep caries near or involving the pulp, early endodontic changes, or localized soft tissue inflammation affecting multiple teeth, without systemic effects); and severe (extensive tissue damage with pulp necrosis, dentoalveolar abscess, fistula formation, or systemic signs such as fever or facial swelling). This three-level framework was used to reflect the range from simple dental decay to neglected conditions that may have systemic effects, helping to stratify cases for both clinical and public health reasons.

Child-level socio-demographic variables—including sex, residential environment (urban vs. rural), birth order within the family, and the child’s level of formal education at the time of assessment—were collected as described previously [15].

### 2.4. Statistical Analysis

All statistical procedures were conducted using IBM SPSS Statistics (version 20.0; IBM Corp., Armonk, NY, USA) along with Microsoft Excel 2013 (version 15.0; Microsoft Corporation, Redmond, WA, USA) for data management and initial descriptive analyses. Quantitative variables were first assessed for distributional normality using the Shapiro–Wilk test and then summarized as means with standard deviations. Categorical variables were presented as absolute frequencies and corresponding percentages.

Between-group comparisons of continuous variables were performed using the independent-samples Student’s *t*-test for normally distributed data or the Mann–Whitney U test and Kruskal–Wallis H test for non-normal data. Post hoc pairwise comparisons of quantitative variables were conducted with the Dunn–Bonferroni procedure. Associations between categorical variables were analyzed using Fisher’s Exact Test, supplemented by Bonferroni-adjusted Z-tests for detailed interpretation of pairwise differences. Correlational analyses between ordinal variables were conducted using Spearman’s rank correlation coefficient (ρ).

To identify independent predictors while accounting for the interrelationships among the explanatory variables, two multivariable binary logistic regression models were constructed, using non-adherence to the dental treatment plan and lesion severity as the respective outcomes. Each model included rural residence, low family income, low caregiver educational attainment, caregiver substance use, and disrupted family structure as predictors. Multicollinearity was evaluated using the variance inflation factor (VIF), with all values below 2. Adjusted odds ratios (aOR) with 95% confidence intervals were reported. The significance threshold was set at *p* ≤ 0.05 for all analyses.

### 2.5. Use of Artificial Intelligence Assistance

During the preparation of this manuscript, the authors used Claude (version Opus 4.8; Anthropic, San Francisco, CA, USA; claude.ai), a large language model (LLM), as a writing assistance tool. Specifically, Claude was used to: (1) assist with academic language editing, rephrasing, and stylistic refinement of all manuscript sections, including the Introduction, Materials and Methods, Results, Discussion, and Conclusions; (2) support the structural organization and formatting of the manuscript in accordance with the requirements of the target journal; and (3) assist in drafting submission-related materials, including the cover letter. The AI tool was not used for data collection, statistical analysis, data interpretation, or clinical decision-making. All AI-assisted content was critically reviewed, verified for accuracy, and approved by all authors. The authors retain full responsibility for the scientific content, conclusions, and integrity of this publication, in accordance with the guidelines of the Committee on Publication Ethics (COPE) and the editorial policies of MDPI.

## 3. Results

### 3.1. Characteristics of the Children Included in the Study

The study involved 333 children, with 176 (52.9%) males and 157 (47.1%) females, showing a mostly uniform sex distribution with a slight male majority (Table 1). The average age of the children was 8.75 ± 3.29 years, with a median age of 8 years, and a range from (4–17). Regarding residential areas, most neglected children (*n* = 272; 81.7%) were from rural areas, while the remaining 18.3% lived in urban areas.

**Figure 1:**
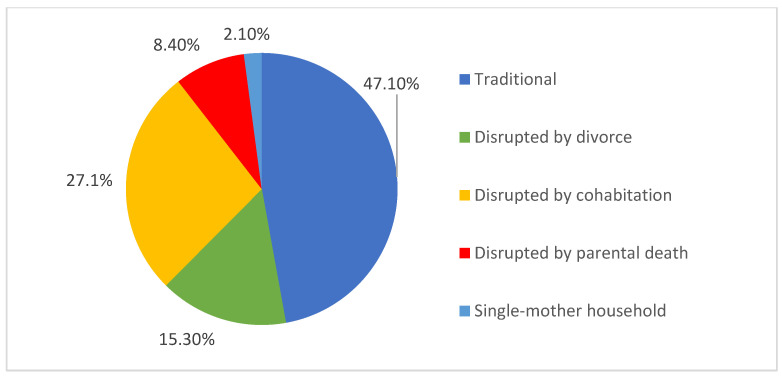
Distribution of study participants by family type.

Figure 1 illustrates the distribution of children according to family type. The majority of children came from traditional families (47.1%), followed by those from disrupted families due to cohabitation (27.1%), disrupted families due to divorce (15.3%), disrupted families due to parental death (8.4%), and single-mother households (2.1%).

### 3.2. Characteristics of the Caregivers (Parents/Guardians)

The data presented in Table 2 reveal that most caregivers were aged between 21 and 40 years, making up 93.1% of the total sample. About 52.0% of caregivers reported not using harmful substances; however, 47.1% indicated alcohol consumption, and 4.8% reported using illicit drugs.

### 3.3. Association Between Caregiver Characteristics—Education and Residential Area

Fisher’s Exact Test revealed a significant relationship between the child’s residential area and the caregiver’s educational level (*p* < 0.001). Post hoc Z-tests with Bonferroni correction showed that caregivers with no formal education (43.4% vs. 26.2%) or who completed primary education (14.7% vs. 0.0%) were more likely to neglect children from rural areas, whereas caregivers with upper secondary education (37.7% vs. 5.5%) or higher education (4.9% vs. 0.0%) were more often associated with neglected children in urban areas (Table 3).

### 3.4. Association Between Family Income and Treatment Plan Adherence

With respect to family income, 297 (89.2%) of children came from low-income households (monthly income: 0–2692 RON), while 36 (10.8%) came from middle-income families (3176–6452 RON). A statistically significant association was identified between family income and adherence to the dental treatment plan (*p* = 0.039). Non-adherence to treatment was more prevalent among low-income families (85.5% vs. 72.2%), whereas adherence to treatment was more frequent among middle-income families (27.8% vs. 14.5%) (Table 4).

### 3.5. Association Between Child Educational Level and Family Income

Regarding the child’s educational level and family income, a statistically significant association was also found (Fisher’s Exact Test, *p* < 0.001). Post hoc Z-tests with Bonferroni correction showed that children with incomplete primary education were significantly more likely to come from low-income households (49.5% vs. 19.4%), while children with incomplete lower secondary education (22.2% vs. 3.4%) or incomplete upper secondary education (13.9% vs. 0.7%) were more often associated with middle-income families (Table 5).

### 3.6. Association Between Caregiver Drug Use and Child Age

A statistically significant difference in the age of neglected children based on caregiver drug use status was found (*p* = 0.014). Children neglected by drug-using caregivers were significantly older (median: 12 years, IQR: 7.5–14) compared to those neglected by non-using caregivers (median: 8 years, IQR: 6–10) (Table 6).

The data presented in Table 7 show the distribution of children by residential area and caregiver drug use. A significant association was also identified between residential area and caregiver drug use (Fisher’s Exact Test, *p* < 0.001), with neglected children whose caregivers were drug users more frequently coming from urban areas (18% vs. 1.8%) (Table 7).

To account for the interrelationships among the socio-behavioral variables, two multivariable logistic regression models were fitted (Table 8). In the model for non-adherence to the treatment plan, none of the included predictors reached statistical significance after mutual adjustment, indicating that no single variable acted as an independent predictor of non-adherence within this cohort. In the model for lesion severity, caregiver substance use emerged as the only significant independent risk factor (aOR = 1.95; 95% CI: 1.11–3.42; *p* = 0.021), with children of substance-using caregivers showing approximately twice the odds of presenting moderate-to-severe lesions. Low caregiver educational attainment was inversely associated with severity (aOR = 0.48; 95% CI: 0.26–0.87; *p* = 0.017); given the observational design and the interrelated nature of the predictors, this finding should be interpreted with caution and not as a genuinely protective effect.

## 4. Discussion

The statistical analysis of children’s distribution by sex showed a relatively balanced gender profile, with a slight male predominance: 52.9% of neglected children were male and 47.1% were female. This almost equal distribution suggests that dental neglect is not significantly dependent on sex; instead, it appears to be more closely related to the quality of parental supervision and oral health education within the family. The slight overrepresentation of boys may partly be due to the earlier maturation and increased esthetic awareness seen in girls as they near adolescence, which could lead to more attention to personal appearance, including dental hygiene. This pattern has been observed in broader pediatric oral health research, where female children and adolescents tend to have better oral hygiene habits than males [15]. Nevertheless, the results of this study highlight that family environment and parental behaviors are more strongly associated with dental neglect, regardless of the child’s sex.

The mean age of the children included in the study was 8.75 ± 3.285 years, with a median of 8 years and an age range from 4 to 17 years. This distribution aligns closely with findings reported in previous research. A 2015 child protection evidence report documented a similar age range of 15 months to 15 years among cases of dental neglect [20]. Likewise, Zins et al. (2019) found that 95% of children identified as victims of dental neglect were under 10 years old [21], supporting the idea that younger children are especially vulnerable because they depend entirely on caregivers for oral health. Several other researchers have also identified preschool-aged children as a high-risk group for dental neglect [14,22,23,24], likely due to the crucial role of parental involvement during early dental development. The concentration of cases in the younger age groups in this study emphasizes the need for early detection and preventive measures, especially for families with children aged four to ten.

The majority of the caregivers identified in this study fell within the 21–40 year age group (93.1%), indicating they are mostly young adults. Regarding education, 40.2% were illiterate, and 20.1% had only completed lower secondary schooling (gymnasium). These data highlight a population with limited life experience and low educational levels—factors often linked to poor parenting practices and insufficient oral health knowledge. Parents are the main role models for their children’s health behaviors, especially when it comes to daily oral hygiene routines, which children tend to learn by watching and copying their parents [5]. When parents do not practice good oral hygiene, their children are significantly more likely to develop similar habits, including not brushing teeth regularly. Parental education level is a key factor influencing oral health literacy and, consequently, dental neglect [23]. Low education levels may limit parents’ understanding of the impacts of poor oral hygiene and may reduce the chances of seeking timely professional dental care for their children. These findings are consistent with global studies showing that higher parental education is linked to lower rates of untreated dental cavities and dental neglect in children [25,26].

Regarding the health status of the participating caregivers, 67.6% were reported to be in generally good health; however, 47.1% were identified as alcohol consumers—a notably high proportion. Alcohol consumption was linked to 12.3% of cases in the study by Taillieu et al. (2021) [27], which is a much lower rate than in the current cohort. This difference may reflect regional variations in substance use or differences in how cases are identified. Parental alcohol and/or drug use is a significant predisposing factor for child neglect as these substances have been associated with reduced caregiver cognitive function, emotional availability, and sense of responsibility. The sedative and disinhibiting effects of alcohol, which depend on the dose, the individual’s mental state, and personality traits, may be associated with lethargy and detachment, which may contribute to disengagement from daily caregiving tasks, including supervising their child’s oral hygiene [28]. This pattern of escapism and avoidance makes children especially vulnerable to neglect in multiple areas, with dental care often being overlooked. The high rate of alcohol use among parents/guardians in this study calls for increased attention from child protection services and dental professionals, who are well placed to identify and refer at-risk families.

Concerning illicit drug use among study caregivers, a notable finding was that children neglected by drug-using caregivers were significantly older (median age range: 7.5–14 years) compared to those neglected by non-using caregivers (median age range: 6–10 years), a difference that was statistically significant (Mann–Whitney U test, *p* = 0.014). This may suggest that the negative effects of parental drug use on child supervision and dental care-seeking become more evident as children grow older and require more complex dental management. Alternatively, older children of drug-dependent parents might have experienced extended periods of neglect before this was recognized by healthcare professionals, due to the insidious nature of addiction-related parental dysfunction. Substance use disorders have been widely linked to child neglect in the literature [29], and the dental setting can serve as a critical point for identifying these vulnerable children.

Low family income emerged as a significant predisposing factor in the present study, with 89.2% of neglected children coming from low-income households and only 10.8% from families with a median income. This finding aligns with a substantial body of research documenting a strong link between low socioeconomic status and poor oral health outcomes in children [29,30,31,32,33], as well as inadequate or missing oral hygiene practices [34,35]. Children from economically disadvantaged families show a higher prevalence of dental caries, especially early childhood caries, compared to children from higher-income households [31,36]. Dietary factors, such as frequent consumption of sugar-laden foods and prolonged use of pacifiers or nursing bottles, which are more common among lower socioeconomic groups, are closely associated with the development of carious lesions [32,37]. Financial limitations may also limit access to professional dental care, preventive services, and oral health education, which may be associated with an increased risk of dental neglect. These findings emphasize the need for targeted public health strategies that address the link between poverty and oral health, including subsidized dental services and community-based preventive programs for low-income families.

Analysis of the relationship between place of residence, the educational level of the caregivers, and the occurrence of dental neglect revealed a statistically significant interaction (Fisher’s exact test, *p* < 0.001). Illiterate parents (43.4% vs. 26.2%) and those who had only completed primary education (14.7% vs. 0%) were more frequently associated with neglect among children living in rural areas, whereas caregivers with secondary (37.7% vs. 5.5%) or higher education (4.9% vs. 0%) were more frequently associated with neglect among children from urban environments. This pattern may reflect differences in educational attainment across geographic areas, as well as the higher concentration of socially disadvantaged families in rural regions with limited access to dental services and health education resources. The impact of geographic remoteness as a barrier to dental care has been confirmed in numerous studies, with rural children consistently showing higher rates of untreated dental disease compared to their urban counterparts (39) [38]. Furthermore, family income was found to significantly influence children’s educational level: in low-income families, children more often had incomplete primary education (49.5% vs. 19.4%), while in middle-income families, children more frequently had incomplete secondary (22.2% vs. 3.4%) or upper secondary education (13.9% vs. 0.7%) (Z-tests with Bonferroni correction). These findings collectively highlight the complex interaction between socioeconomic disadvantages, geographic isolation, and educational deprivation as interconnected risk factors for dental neglect.

Non-adherence to the proposed dental treatment plan was observed in the vast majority of cases, with 84.1% of parents or guardians failing to attend scheduled follow-up appointments. Dental neglect is further confirmed when a parent or guardian does not return with the child for subsequent appointments, despite being informed of the child’s dental problems, the severity of the lesions, the planned treatment, the potential long-term consequences of non-treatment, and the availability of pain-free treatment under anesthesia [39]. The extent of non-adherence documented in this study is significantly higher than figures reported in the United Kingdom, where a non-attendance rate of 11–12% was found among a cohort of 100 children [40]. This difference likely reflects the combined influence of low educational levels, financial constraints, and geographic remoteness common in the study population. Adherence to the dental treatment plan was notably higher among children from urban areas (26.2% vs. 13.6%, Fisher’s exact test, *p* = 0.020) and among those from median-income families (27.8% vs. 14.5%, Pearson Chi-Square, *p* = 0.039). These findings suggest that proximity to dental services, better living standards, and increased access to health education through mass media may be associated with lower levels of reduced dental neglect. The high rate of non-adherence observed in this study underscores the importance of active follow-up strategies and community outreach programs, especially for families living in rural and low-income areas.

Family structure was identified as an additional factor associated with dental neglect. More than half of the children in the study (52.9%) came from disrupted family environments, with cohabitation (concubinage) being the most common form of family disorganization (27%). Disrupted family structures—especially those characterized by cohabitation—may be associated with disengagement and less investment in children’s well-being, particularly when the child is not biologically related to the cohabiting partner. This situation may be linked to weaker emotional attachment and a sense of caregiving responsibility, and to a higher risk of neglect. The current findings align with those of several studies reporting a higher risk of neglect among children raised by single parents or in non-traditional family units [13,20,21,23,41,42,43,44]. The link between family disorganization and child neglect is believed to reflect the cumulative stressors faced by single-parent or cohabiting families, including financial difficulties, social isolation, and reduced parental availability, all of which may be associated with less consistent and adequate caregiving. Dental professionals should be mindful of family structure as a contextual risk factor during clinical assessments, especially when other signs of neglect are evident.

Taken together, the findings of this study identify a consistent profile of risk factors linked to child dental neglect: young, less-educated, and financially disadvantaged parents or guardians, mostly living in rural areas, with high rates of alcohol use and unstable family structures. These factors do not appear to act in isolation but rather tend to co-occur and overlap, collectively contributing to the vulnerability of the affected children. The retrospective cross-sectional analytical design of this study enabled systematic documentation of these associations within a real-world clinical setting. These observations were further supported by multivariable analysis. After mutual adjustment, caregiver substance use remained the only independent predictor of greater lesion severity, whereas none of the variables independently predicted treatment non-adherence. This pattern indicates that the bivariate associations observed for several socio-economic factors are likely interrelated rather than independent, reinforcing the view that dental neglect arises from a cluster of co-occurring rather than isolated risk factors.

Beyond confirming established risk associations, these findings have direct implications for everyday dental practice, particularly in settings comparable to the present one. Rather than a general appeal to safeguarding, several concrete measures can be recommended. First, dental teams should receive periodic, structured training in recognizing the clinical and contextual indicators of dental neglect, including the combination of clinical signs and caregiver-related factors applied in this study. Second, repeated missed appointments should be managed through a formal protocol that reframes them from “did not attend” to “was not brought,” ensuring that each missed visit is documented chronologically and prompts active follow-up rather than passive discharge [39]. Third, structured caregiver history and a standardized record of treatment-seeking behavior should be incorporated into routine pediatric dental assessment, so that persistent unmet needs are identified despite caregiver awareness and professional recommendation [14,20]. Fourth, a clear referral threshold should be defined: in accordance with Romanian Law No. 272/2004 on the protection and promotion of the rights of the child, any professional working directly with a child who suspects abuse or neglect is legally obliged to notify the local General Directorate for Social Assistance and Child Protection (DGASPC) or the national child helpline (single European number 119) [45]. Within a clinical pathway, this threshold can be operationalized as persistence of unmet oral healthcare needs after the caregiver has been informed of the diagnosis, the planned treatment, and the consequences of non-treatment, and after reasonable barriers to access have been addressed. Finally, given the multifactorial nature of neglect, suspected cases should be managed through interdisciplinary collaboration involving pediatricians, family physicians, and social services, rather than by the dental team alone [4,14].

Regarding this study, some limitations must be acknowledged, including the dependence on clinical records for data collection, which may lead to incomplete or inconsistently documented information, and the geographic focus of the sample. As all participants were recruited from a single Romanian county, through one municipal hospital and an associated private dental practice, the external validity of the findings is limited. The results reflect the specific socio-economic conditions of this region and the characteristics of the local healthcare system, including patterns of access to dental services. Consequently, these findings should be generalized with caution to other regions or countries, particularly when considering broader international implications, where socio-economic contexts and healthcare delivery systems may differ substantially. In addition, no validated dental neglect-specific screening instrument was routinely applied during clinical care, and formal inter-examiner reliability statistics were not prospectively recorded due to the retrospective study design; nevertheless, case identification relied on a structured clinician-based assessment derived from AAPD criteria, supported by caregiver history, documented treatment behavior, and consensus evaluation between calibrated examiners. Future prospective studies that include standardized sociodemographic assessments and validated neglect screening tools are needed to better understand the associations between parental socio-behavioral traits and child dental neglect, and to support the development of targeted preventive strategies.

## 5. Conclusions

The study identifies a clear socio-behavioral profile associated with dental neglect in children from Bihor County, predominantly affecting younger children from rural, low-income families in the context of young caregivers with low educational attainment, alcohol or substance use, and unstable family structures. The identified risk factors, namely poverty, geographic isolation, limited education, and family dysfunction, frequently co-occur and appear closely interrelated rather than acting in isolation. The dental setting represents a critical point for early identification, and the active involvement of dental professionals in child protection protocols is both a professional and ethical responsibility. The study’s limitations call for future prospective research using standardized tools and broader sampling, to support the development of effective prevention strategies.

## Figures and Tables

**Table 1:** Distribution of children by sex, residential area, formal education and psychological status.

Variable		No.	Percentage
Sex	Female	157	47.1%
	Male	176	52.9%
Environment	Rural	272	81.7%
	Urban	61	18.3%
Child’s Formal Education	Without studies	24	7.2%
	Preschool level	95	28.5%
	Primary studies not completed	154	46.2%
	Completed primary studies	26	7.8%
	Unfinished secondary school	18	5.4%
	Completed secondary school	9	2.7%
	Unfinished high school education	7	2.1%
Psychological/Behavioral status	Healthy	263	76%
	Drug Users	56	16.8%
	Intellectual disability	24	7.2%

**Table 2:** Distribution of caregivers by age, substance use, educational level, and health status.

Variable		No.	Percentage
Age	<21 years	7	2.1%
	21–40 years	310	93.1%
	41–60 years	16	4.8%
Harmful Substance Use	No Substance Use	162	48.6%
	Drug User	14	4.2%
	Alcohol User	157	47.1%
Caregivers Formal Education	No formal education	134	40.2%
	Incomplete primary education	51	15.3%
	Complete primary education	40	12.0%
	Complete lower secondary education	67	20.1%
	Upper secondary education	38	11.4%
	Higher education	3	0.9%
Health status	Clinically healthy	225	67.6%
	Chronic disease(s) present	108	32.9%

**Table 3:** Distribution of children by caregiver educational level and residential area.

Caregiver Education/ Residential Area	Rural		Urban		*p* *
	No.	Percentage	No.	Percentage	
No formal education	118	43.4%	16	26.2%	<0.001
Incomplete primary education	44	16.2%	7	11.5%	
Completed primary education	40	14.7%	0	0.0%	
Complete lower secondary education	55	20.2%	12	19.7%	
Upper secondary education	15	5.5%	23	37.7%	
Higher education	0	0.0%	3	4.9%	

* Fisher’s Exact Test; post hoc Z-tests with Bonferroni correction.

**Table 4:** Distribution of children by family income and treatment plan adherence.

Family Income/ Treatment Plan	Low Income		Middle Income		*p* *
	No.	Percentage	No.	Percentage	
Non-adherence to the treatment plan	254	85.5%	26	72.2%	0.039
Adherence to the treatment plan	43	14.5%	10	27.8%	

* Pearson Chi-Square Test.

**Table 5:** Association between family income and children’s educational level.

Child Education Level/ Family Income	Low Income		Middle Income		*p* *
	No.	Percentage	No.	Percentage	
Without studies	23	7.7%	1	2.8%	<0.001
Preschool level	83	27.9%	12	33.3%	
Primary studies not completed	147	49.5%	7	19.4%	
Completed primary studies	25	8.4%	1	2.8%	
Unfinished secondary school	10	3.4%	8	22.2%	
Completed secondary school	7	2.4%	2	5.6%	
Unfinished high school education	2	0.7%	5	13.9%	

* Fisher’s Exact Test; post-hoc Z-tests with Bonferroni correction.

**Table 6:** Comparison of child age by caregiver drug use status (Mann–Whitney U Test).

Caregiver Drug Use	Mean ± SD (Years)	Median (IQR) (Years)	Mean Rank	*p* *
Absent	8.65 ± 3.218	8 (6–10)	163.61	0.014
Present	11 ± 3.847	12 (7.5–14)	223.59	

* Mann–Whitney U Test.

**Table 7:** Distribution of Children by Residential Area and Caregiver Drug Use.

Caregiver Drug Use/ Residential Area	Rural		Urban		*p* *
	No.	Percentage	No.	Percentage	
No substance use	267	98.2%	50	82.2%	<0.001
Drug use	5	1.8%	11	18%	

* Fisher’s Exact Test.

**Table 8:** Multivariable logistic regression models for treatment non-adherence and lesion severity.

Predictor	Non-Adherence aOR (95% CI)	*p*	Severity aOR (95% CI)	*p*
Rural residence	1.97 (0.82–4.72)	0.127	1.05 (0.43–2.54)	0.911
Low family income	0.91 (0.29–2.84)	0.875	1.41 (0.46–4.37)	0.548
Low caregiver education	1.76 (0.86–3.59)	0.119	0.48 (0.26–0.87)	0.017
Caregiver substance use	1.25 (0.67–2.32)	0.483	1.95 (1.11–3.42)	0.021
Disrupted family structure	1.63 (0.89–2.99)	0.115	1.20 (0.70–2.05)	0.516

aOR = adjusted odds ratio; CI = confidence interval. Maximum VIF = 1.86.

## Data Availability

The data presented in this study are available on request from the corresponding author. The data are not publicly available due to privacy reasons.
